# Modulation of Bacterial Type III Secretion System by a Spermidine Transporter Dependent Signaling Pathway

**DOI:** 10.1371/journal.pone.0001291

**Published:** 2007-12-12

**Authors:** Lian Zhou, Jing Wang, Lian-Hui Zhang

**Affiliations:** 1 Institute of Molecular and Cell Biology, Singapore, Singapore; 2 Department of Biological Sciences, National University of Singapore, Singapore, Singapore; Columbia University, United States of America

## Abstract

**Background:**

Many gram-negative bacterial pathogens employ Type III secretion systems (T3SS) to inject effector proteins into host cells in infectious processes.

**Methodology/Principal Findings:**

By screening a transposon mutant library of *P. aeruginosa*, we found that mutation of *spuDEFGH*, which encode a major spermidine uptake system, abolished the expression of the *exsCEBA* operon that codes for key T3SS regulators under inducing conditions (low calcium). Whole genome microarray analysis revealed that inactivation of the spermidine uptake system significantly decreased the transcriptional expression of most, if not all, T3SS genes. Consistently, the spermidine uptake mutants showed decreased expression of the T3SS genes in responding to host cell extract and attenuated cytotoxicity. Furthermore, exogenous addition of spermidine to the wild type strain PAO1 enhanced the expression of *exsCEBA* and also the effector protein genes.

**Conclusion/Significance:**

Cumulatively, these data have depicted a novel spermidine transporter-dependent signaling pathway, which appears to play an essential role in modulation of T3SS expression in *P. aeruginosa*.

## Introduction

Type III secretion system (T3SS) is a key virulence determinant in a wide range of animal and plant pathogens. This protein secretion and delivery system specifically facilitates the translocation of bacterial effector proteins into eukaryotic host cells [1], [2], and initiates a sophisticated “biochemical cross-talk” between pathogen and host [3]. T3SS plays diverse roles in host-pathogen interactions, such as promoting bacteria internalization in mammalian cells [4], [5], induction of macrophage apoptosis [6], inhibition of phagocytosis by changing macrophage actin structures [7], and generation of pores in host cells [8].

*Pseudomonas aeruginosa* is an important opportunistic human bacterial pathogen that causes serious infections in immuno-compromised individuals [9], [10]. The pathogen deploys a range of virulence determinants to enhance its competitive advantages against its host. Notably, most clinical isolates of *P. aeruginosa* use T3SS as a specialized mechanism to evade phagocytosis and facilitate infections by translocating effector proteins, including ExoS, ExoT, ExoU and ExoY, into host cells [11]–[15]. Among them, ExoS and ExoT are bi-functional proteins, possessing a GTPase-activating activity at the N-terminus and an ADP-ribosyltransferase domain within the C-terminus [16]–[19]; ExoY is an adenylate cyclase whose activity is associated with morphological changes of epithelial cells [15], [20]; and ExoU is an acute cytotoxin with phospholipase activity [13], [20].

About 43 T3SS genes have been identified in *P. aeruginosa* [21], but not all isolates contain the same repertoire of effector genes. For example, cytotoxic *P. aeruginosa* isolates possess *exoU*, whereas non-cytotoxic isolates lack this gene [22]. Despite this variation, the general T3SS regulatory mechanism appears to be highly conserved among *P. aeruginosa* isolates. Expression of T3SS can be induced by three types of environmental signals, i.e., (i) limitation of calcium, (ii) serum, and (iii) contact with eukaryotic cells [21]–[23]. The transcriptional expression of these T3SS genes are coordinated by its master regulator ExsA encoded by the *exsCEBA* operon, which activates the T3SS system by binding to the promoter motif of known T3SS genes [21], [22]. Under non-inducible conditions, such as high calcium, the type III secretion channel is closed and the negative regulator ExsE forms a complex with the positive regulator ExsC in cytoplasm, which consequently allows the repressor ExsD to disable ExsA by formation of a heterodimeric complex. In contrast, when calcium is limited, ExsE is secreted out of bacterial cells through the type III secretion channel, which leaves ExsC to interact with ExsD and releases ExsA to activate T3SS [24], [25]. Evidence also suggests that the expression and function of ExsA are modulated by several upstream regulatory proteins, including the cAMP-dependent transcription factor Vfr [26], the two-component hybrid regulators RtsM and RetS [27], [28]. Albeit little is known about the underlying mechanisms by which these regulators respond to environmental cues, it becomes clear that regulation of T3SS is in general through modulating the activity of the master regulator ExsA whose expression is under the control of the *exsCEBA* promoter [21], [22].

In this study, we set to explore the potential signaling pathways which modulate the expression of the *exsCEBA* operon. For this purpose, we fused the reporter gene *lacZ* gene to the *exsCEBA* promoter and integrated the gene cassette into the chromosome of *P. aeruginosa*. Transposon mutagenesis of the resulted reporter strain showed that mutation of the *spu* genes encoding a major spermidine uptake system significantly decreased the expression of the *exsCEBA* operon. By using whole genome microarray analysis, we found that deletion of the spermidine system significantly decreased the expression of 37 T3SS genes. We further showed that mutation of the spermidine transporter abolished the host cell-induced the T3SS gene expression, demonstrating the essential role of the transporter in signal modulation of the T3SS system.

## Results

### Induction of *exsCEBA* Required the Major Spermidine Uptake Transporter Encoded by *spuDEFGH*

**Figure 1:**
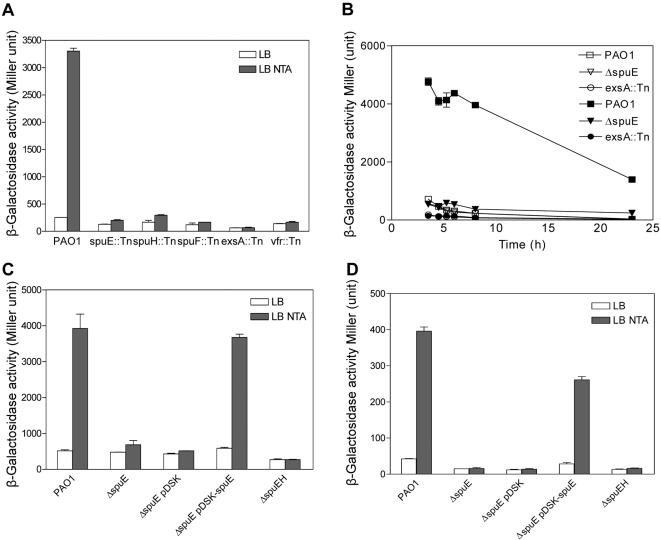
Mutation of the genes encoding the SpuDEFGH transporter decreased the expression of T3SS genes. (A) The *exsCEBA* expression patterns of five transposon mutants under normal and low calcium condition. (B) Time course analysis of the *exsCEBA* expression in strain PAO1 and in the spermidine transporter mutants. (C) Deletion of the transporter abolished the low calcium-induced expression of *exsCEBA*. (D) Deletion of the transporter abolished the low calcium-induced expression of *exoT*. Bacteria were grown in LB medium with or without 7.5 mM NTA, indicated by solid and open bar/symbol, respectively. The experiment was repeated twice and the data were the means of three replicates.

The promoter-*lacZ* fusion reporter systems of several key T3SS genes, including the master regulator gene *exsA*, the effector genes *exoS* and *exoT*, have been previously exploited for screening and characterization of regulatory genes [26], [29]. To identify the genes that regulate the expression of T3SS in *P. aeruginosa*, we used a similar approach by fusing the reporter gene *lacZ* to the promoters of *exoT (pT)* and *exsCEBA (pC),* respectively. The fusion genes *pC-lacZ* and *pT-lacZ* were then integrated separately at the *attB* site of the strain PAO1 to generate the report strains PAO1pTlacZ and PAO1pClacZ following a previously described method [30]. After screening for about 15,000 transposon insertion mutants of strain PAO1pClacZ on the LB plates containing NTA and X-gal, nine colonies showing reduced β-galactosidase activity were further analyzed. Arbitrary PCR and DNA sequencing analysis revealed that in three mutants the transposon was inserted in the coding regions of *exsA* and *vfr*, respectively, which are known T3SS regulatory genes [26], [31]. The analysis also identified six other genes whose association with T3SS had not been described previously. Among these new genes, *spuE*, *spuF* and *spuH*, together with the *spuD and spuG* in the same operon (Figure S1), encode a major spermidine uptake transporter in *P. aeruginosa* [32]. Intrigued by the possibility that the transporter might be involved in signaling modulation of the T3SS gene expression, these *spu* mutants were selected for further characterization in this study. *In silico* analysis indicated that *spuD* and *spuE* encode the periplasmic binding protein components, *spuF* encodes the ATPase component, and *spuG* and *spuH* encode the inner membrane permease components for an ABC-type transport system. Quantitative analysis of the *lacZ* activity showed that the transcription level of *exsCEBA* increased by about 8 fold after 4 h growth under T3SS-inducible conditions, whereas no obvious increase in *exsCEBA* expression was detected in all the three *spu* transposon mutants upon calcium depletion (Figure 1A). As expected, mutation of the known T3SS regulatory genes *vfr* and *exsA* also abolished the inducible expression of *exsCEBA* (Figure 1A).

In order to confirm the roles of the *spu* genes, the *spuE* and *spuEFGH* in-frame deletion mutants were generated in the wild type strain PAO1 (Figure S1). The transcriptional fusion genes *pC-lacZ* and *pT-lacZ* were integrated respectively into the genomes of deletion mutants ΔspuE and ΔspuEFGH in the same way as described for the strain PAO1pClacZ. Similar to the three *spu* transposon mutants, deletion of *spuE* or *spuEFGH* abrogated the inducible expression of *exsCEBA* and *exoT* upon calcium depletion (Figure 1C, 1D). The time course study further confirmed that deletion of *spuE* did not affect bacterial growth (Figure S2), but disabled the low-calcium induced *exsCEBA* expression throughout the growth (Figure 1B). Moreover, *in trans* expression of *spuE* in the deletion mutant ΔspuE restored the calcium-depletion inducible expression pattern of *exsCEBA* and *exoT* (Figure 1C, 1D). Taken together, these data suggest the critical role of spermidine transporter in modulating the expression of these T3SS genes.

### Null Mutation of the Spermidine Transporter Down-regulated the Transcription of T3SS genes

**Figure 2:**
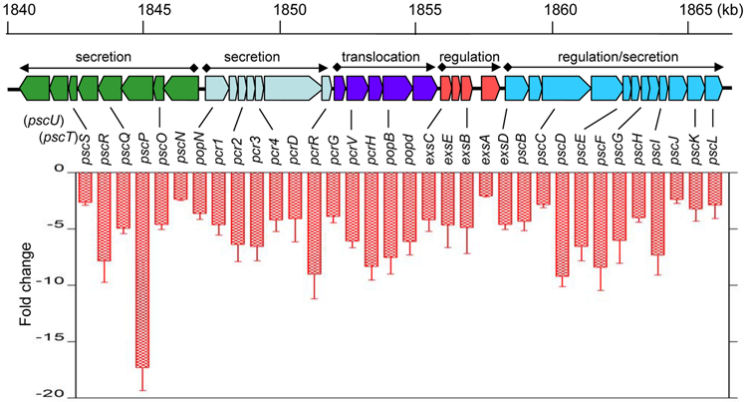
Inactivation of the spermidine transporter led to decreased transcription of T3SS genes. Top panel shows the relative location and size of the genes required for secretion, translocation and T3SS-related regulatory activities, which are located contiguously on the chromosome within five operons (based on the genome sequence data of strain PAO1, http://prodoric.tu-bs.de/gsearch.php). The ExsA-dependent promoter and transcriptional direction are indicated by diamond and arrow, respectively. Bottom panel indicates the transcriptional fold changes of these T3SS genes in the deletion mutant ΔspuE in comparison with the wild type strain PAO1. The transcriptional changes of three effector genes, i.e., *exoT*, *exoY*, and *exoS*, which are located elsewhere on the chromosome, were described in the text.

To determine the scope of influence, we examined the global gene expression profiles of strain PAO1 and the mutant ΔspuE under calcium-depletion conditions using whole genome microarray. In *P. aeruginosa*, thirty six T3SS genes involved in secretion, translocation and regulation are located in a chromosomal locus about 26 kb in size [21]. These T3SS genes constitute five ExsA-dependent operons (Figure 2). The microarray results showed that the transcriptional expression levels of 34 out of the 36 genes at this locus were significantly decreased (≧2-fold) in the deletion mutant (Figure 2). The affected genes include those encoding the T3SS secretion and translocation (*pscNOPQRS*, *popN-pcr1234DR*, *pcrGVH-popBD, pscBCDEFGHIJKL*), and regulation (*exsCEBA*, *exsD*). The remaining two less significantly affected T3SS genes at the same locus were *pscU* and *pscT* (Figure 2), which were moderately down-regulated by about 1.6-fold in the mutant. In addition, the transcriptional expressions of three T3SS effector genes (*exoT*, *exoY*, *exoS*), which are located elsewhere on the chromosome, were markedly decreased by 7.5-, 4.8- and 8.3-fold, respectively, in the spermidine transporter mutant. The null mutation of the spermidine transporter also increased the transcript level of six uncharacterized genes by about 2.3- to 12.1-fold, including one probable transcriptional regulator (PA2432, 5.3-fold), two probable hydrolases (PA1202, 12.1-fold; PA2698, 2.5-fold), and three hypothetical proteins (PA0572, 3.2-fold; PA1203, 4.3-fold; PA2433, 2.3-fold). Interestingly, this SpuDEFGH transporter-dependent signaling system appears to be highly specific for positive regulation of T3SS, as the mutation did not decrease the transcriptional expression of other genes under the assay conditions used in this study.

### Deletion of *spuE* Impaired the Production and Secretion of the T3SS Effector ExoS

**Figure 3:**
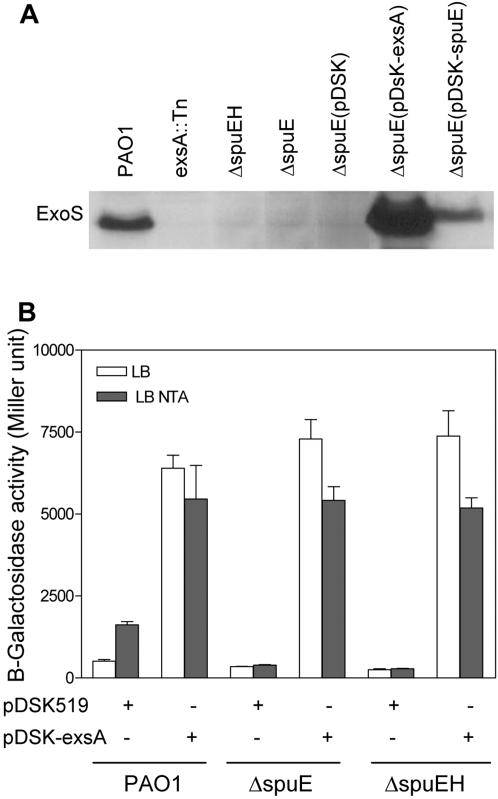
*In trans* expression of *exsA* restored the T3SS gene expression and effector production in the spermidine transporter mutants. (A) Inactivation of the spermidine transporter inhibited the production of the type III effector protein ExoS but the phenotype was rescued by expression of *exsA*. Bacterial cultures were grown overnight in LB medium with or without 7.5 mM NTA and the extra-cellular proteins in supernatants were collected by trichloroacetic acid precipitation and separated by 10% SDS-PAGE. The proteins were transferred onto nitrocellulose membrane and blotted with anti-ExoS antibody (upper panel). (B) Overexpression of *exsA* in the transporter mutants resulted in a high level expression of the T3SS genes *exsCEBA*. Symbol: “+” and “−” indicate whether the plasmid or expression construct was carried by the bacterial strains. The data were the means of three replicates.

Considering that the activator ExsA is required for the secretion of T3SS effectors [31], we further determined the role of the spermidine uptaking system on secretion of the effector ExoS. No secreted ExoS was detected in the supernatants from ΔspuE and ΔspuEH, as well as the two negative controls including mutant *exsA::Tn* and strain ΔspuE(pDSK) containing the plasmid vector pDSK519. In contrast, ExoS was easily detectable from the culture supernatant of wild type strain PAO1 and the complementation strain ΔspuE(pDSK-spuE) grown under low-calcium conditions (Figure 3A).

Previous studies show that transcription of T3SS genes is coupled to secretion and could be blocked in the absence of type III secretory activity [21]. To determine whether disruption of the spermidine transporter may have a pleiotropic effect on the type III secretion machinery, the master regulator gene *exsA* was cloned under the control of the *lac* promoter in plasimd vector pDSK519 to generate the expression construct pDSK-exsA. Abundant of ExoS effector proteins were detected from the culture supernatant of strain ΔspuE(pDSK-exsA) (Figure 3A), indicating that mutation of the transporter did not affect the functionality of the T3SS secretion machinery. In addition, overexpression of *exsA* in strains ΔspuE(pDSK-exsA), ΔspuEH(pDSK-exsA), and wild type PAO1 resulted in a similar high level of expression of *exsCEBA* with or without NTA (Figure 3B), which seems to preclude the possibility that spermidine may act as a signal ligand of ExsA.

### Exogenous Addition of Spermidine Induced the Expression of T3SS and Secretion of Effector ExoS

**Figure 4:**
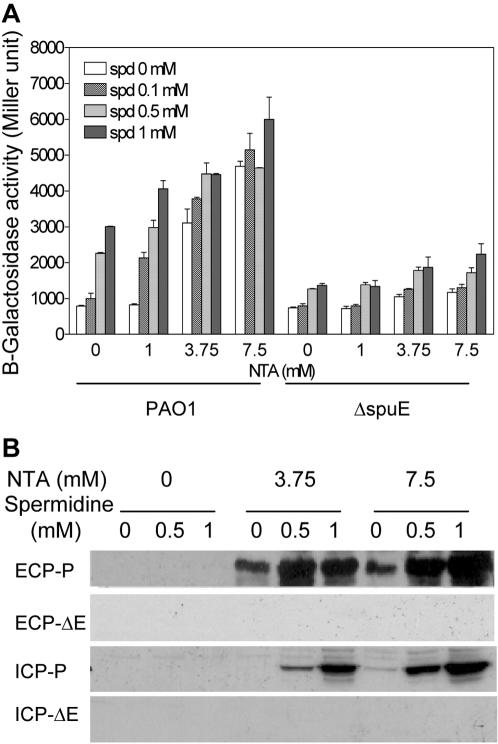
Exogenous spermidine induction of the T3SS system required a functional SpuDEFGH transporter. (A) The *exsCEBA* promoter directed β-galactosidase activity. Results were the means of three replicates. (B) Immunoblotting detection of the effector ExoS. Bacterial cultures were grown in LB medium supplemented with NTA and spermidine as indicated. The extra-cellular proteins (ECP) and intra-cellular proteins (ICP) from strains PAO1 (P) and ΔspuE (ΔE) were separated by 10% SDS-PAGE. The proteins were transferred onto nitrocellulose membrane and blotted with anti-ExoS antibody. The experiment was repeated for at least three times with similar results.

The *spuDEFGH* genes were reported to encode a major ABC-type transporter system for spermidine uptake, and knocking out any of these genes significantly reduces the ability of *P. aeruginosa* to utilize spermidine as the sole source of carbon and nitrogen in a defined minimum medium [32]. This finding and our results suggest that the pathogen may as well use this transport system to take up exogenous spermidine as a novel T3SS activation signal. To test this hypothesis, we added different amounts of spermidine (up to 1 mM) to the LB medium, and monitored the bacterial growth by measuring absorbance at 600 nm and the expression of *exsCEBA* in strains PAO1 and ΔspuE by measuring the β-galactosidase activity encoded by the *pC-lacZ* reporter gene. While supplementation of spermidine had no effect on the growth rate of both PAO1 and ΔspuE (Figure S2), it affected the T3SS gene expression in a dosage-dependent manner. In the absence of NTA, addition of 0.1 mM spermidine to strain PAO1 did not show significant effect, but increasing its concentration to 0.5 and 1 mM enhanced the expression of *exsCEBA* by about 2–3 folds, respectively (Figure 4A). The calcium chelator NTA at a final concentration of 1 mM did not have any effect on *exsCEBA* expression, however, the maximal effect of spermidine was observed under this condition. Addition of 0.1, 0.5 and 1 mM spermidine increased the *exsCEBA* expression by 2- to 4-fold, respectively (Figure 4A). These data indicate that exogenous spermidine induced *exsCEBA* expression in a dose-dependent manner. In contrast, deletion of *spuE* largely attenuated the inducible effect of spermidine (Figure 4A); the low level of induction may suggest the mutant could partially take up exogenous spermidine, agreeable with the previous finding the SpuDEFGH is the major but not the sole spermidine transporter [32]. In the presence of a high concentration of NTA (3.75 and 7 mM), spermidine was still able to induce T3SS gene expression but became less significant than being with low NTA (Figure 4A).

To further study the effect of exogenous spermidine on secretion of T3SS effectors, we used ExoS-specific antibody to detect its presence in bacterial cellular and extracellular fractions. Wild type PAO1 had a readily detectable amount of ExoS in both supernatant and cellular protein fractions when grown under low calcium conditions, and addition of 0.5 or 1 mM of spermidine further increased the ExoS content in both fractions (Figure 4B). On the contrary, no ExoS was detectable from the deletion mutant ΔspuE grown under the same conditions (Figure 4B).

### Spermidine was the Most Effective Polyamine Signal for T3SS Induction

To determine the specificity of potential signal molecules, we compared the activity of a range of polyamines on induction of *exsCEBA* expression in wild type strain PAO1 using ornithine, the precursor of polyamines, as the negative control. The results showed that spermidine displayed the strongest relative signaling activity (100%), followed by spermine (79.0%) and acetyle-spermidine (21.6%), whereas the remaining polyamines including putrescine, cadaverine, and norspermidine were not able to induce T3SS genes at a final concentration of 1 mM (Table 1).

**Table 1:** Effect of polyamines on the expression of *exsCEBA*.

Chemical	Molecular formula	Relative activity (%±SD)
ornithine	C_5_H_12_N_2_O_2_	0
putrescine	NH_2_(CH_2_)_4_NH_2_	6.0±2.7
cadaverine	NH_2_(CH_2_)_5_NH_2_	5.4±6.6
spermine	NH_2_(CH_2_)_3_NH(CH_2_)_4_NH(CH_2_)_3_NH_2_	79.0±13.3
spermidine	NH_2_(CH_2_)_3_NH(CH_2_)_4_NH_2_	100±3.9
norspermidine	(NH_2_CH_2_CH_2_CH_2_)_2_NH	4.2±2.0
acetyl spermidine	CH_3_CH_2_NH(CH_2_)_3_NH(CH_2_)_4_NH_2_	21.6±10.1

### Mutation of Spermidine Transporter Decreased the Host Cell Extract-induced Expression of T3SS genes and Attenuated the T3SS-mediated Cytotoxicity

**Figure 5:**
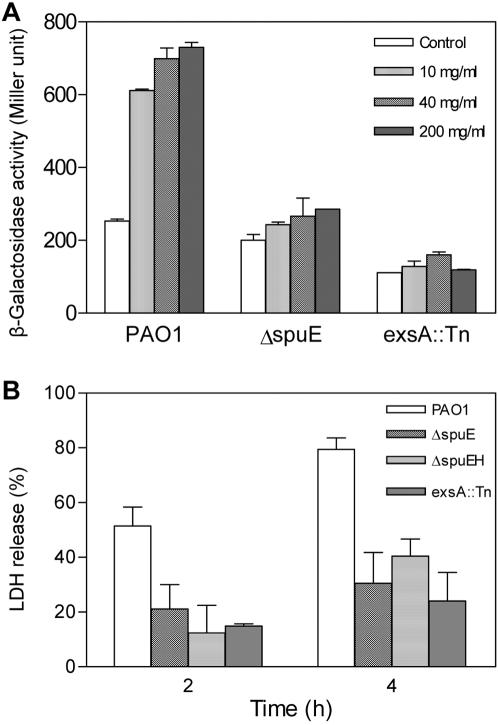
Inactivation of the spermidine transporter reduced the host cell extract-dependent expression of *exsCEBA* and attenuated the T3SS-mediated cytotoxicity. (A) The *exsCEBA* promoter directed β-galactosidase activity after culture of the bacterial cells for 4 h in minimum medium with various amounts of mouse liver extract (indicated as equivalent to liver tissue fresh weight per ml culture). The bacteria grown in the same minimum medium was used as a blank control. (B) Cytotoxicity was assayed by monitoring LDH release by the HeLa cells infected with a MOI of 40–50. Experiments were performed with DMEM medium supplemented with 1% FBS and 2 mM glutamine. The data were the means of at least three replicates.

Given that eukaryotic organisms produce abundant spermidine and other polyamines [33], we speculated that mutation of spermidine uptake system may have significant impact on host cell-induced expression of T3SS genes. To test this hypothesis, we mixed a portion of minimum medium containing various amounts of liver extract with the same volume of the medium containing fresh bacterial cells and monitored the expression of *exsCEBA*. After co-culture for 4 h with liver extract equivalent to 10, 40, and 200 mg of liver tissue per ml of culture mixture, the *exsCEBA* expression in wild strain PAO1 was increased by about 2.5- to 4.5-fold, in comparison with the corresponding blank control of bacterial cells growing in minimum medium without liver extract and the *exsA* mutant that was used as the negative control (Figure 5A). In contrast, mutation of the spermidine transporter substantially decreased the liver extract-induced *exsCEBA* expression (Figure 5A).

To test the biological significance of the SpuDEFGH transporter, we determined the cytotoxicity of strain PAO1 and its derivatives on the human epithelial cell line HeLa. Quantitative determination of lactate dehydrogenase (LDH) release at 2 h and 4 h post infection showed that comparing with the wild type strain PAO1, inactivation of the spermidine transporter by deleting *spuE* or *spuEFGH* significantly decreased the cytotoxicity by up to 60% (Figure 5B).

## Discussion

By engineering a strain with a chromosomally integrated T3SS reporter gene, we conducted a transposon mutagenesis of the bacterial pathogen *P. aeruginosa* to identify the potential genes implicated in signal modulation of the T3SS gene expression. The analysis led to identification of three genes, i.e., *spuE*, *spuF*, and *spuH*, which together with *spuD* and *spuG* encode an ABC-type transporter involved in spermidine uptake [32]. Several lines of evidence supported that this transporter has a prominent role in modulation of the T3SS gene expression. First, mutation of any of the *spuEFH* genes abrogated the calcium depletion-induced expression of the *exsCEBA* genes (Figure 1A). Second, deletion of either *spuE* or *spuEFGH* abrogated the induced expression of the *exsCEBA* and *exoT* genes (Figure 1B, 1C, 1D). Third, deletion of *spuE* significantly decreased the expression of most T3SS genes under inducible conditions and abolished the production of effector proteins (Figure 2, Figure 3A). Furthermore, inactivation of the spermidine transoporter decreased the host cell extract-dependent induction of T3SS system and attenuated the bacterial cytotoxicity (Figure 5A, 5B).

Among the several polyamines tested, only spermidine and spermine were able to induce the expression of T3SS genes with spermidine being the strongest inducer (Table 1), which is agreeable with the previous report that spermidine is the preferred ligand molecule of the SpuDEFGH transporter system [32]. Consistently, deletion of the transporter significantly attenuated the spermidine activity on induction of T3SS genes and abrogated the production of T3SS effector proteins (Figure 4A, 4B). These findings strongly suggest that the SpuDEFGH transporter system is required for influx of exogenous polyamine signals, in particular, spermidine and spermine, into the bacterial cells.

Our data suggest that *P. aeruginosa* may exploit the host-originated signals to trigger the expression of T3SS genes. Spermine is present only in eukaryotic cells whereas spermidine is present in both prokaryotic and eukaryotic cells [32]–[34]. However, we found that deletion of the *speD* and *speE* genes, which encode the key enzymes for spermidine biosynthesis [32], did not have obvious effect on T3SS gene expression in *P. aeruginosa* under the assay conditions used in this study (Figure S3), suggesting that bacterial pathogens may likely respond to the polyamine signals from host organisms. Spermine and spermidine are normally present at millimolar concentrations in plants and animals [33]. The total spermidine concentrations in the rat liver and bovine lymphocyte were estimated to be about 1.15 and 1.33 mM, respectively [33]. The findings that spermidine at about 1 mM can effectively induce the expression of T3SS genes under low-calcium conditions (Figure 4A, 4B) suggest that the bacterial pathogen might sense this *in vivo* signal to trigger the expression of T3SS genes. The notion is greatly strengthened by the observation that deletion of the spermidine transporter significantly reduced the host cell extract-induced expression of T3SS genes and attenuated the T3SS-mediated cytotoxicity of *P. aeruginosa* (Figure 5A, 5B).

It becomes highly intriguing how spermidine and spermine could modulate the expression of T3SS genes. Previous studies have shown that cationic polyamines could participate in many cellular processes via binding to DNA, RNA, nucleotide signals and other acidic substances [33]. Our finding that disruption of the spermidine transporter exclusively down-regulated the T3SS genes seems to suggest a rather specific mechanism by which the polyamine signals act on. Albeit the mechanism is unknown, several lines of evidence indicate that spermidine functions by modulating the transcriptional expression of the master regulator gene *exsA*. Firstly, mutation of the spermidine transporter significantly decreased the transcriptional expression of *exsA* according to transcriptional gene fusion and microarray data (Figure 1A, 1B, 1C; Figure 2). Secondly, *in trans* expression of *exsA* in the transporter mutants restored the ExsA-dependent functions including expression of T3SS genes and production of effector proteins (Figure 3A, 3B), which precludes the possibility that null mutation of the transporter may also affect the functionality of the type III secretory machinery. It is known that interruption of the type III secretory machinery could lead to a feedback inhibition of the transcriptional expression of T3SS genes [21]. In addition to autoregulation by its own product, transcriptional expression of *exsA* is also influenced by other global signaling systems such as quorum sensing, Vfr and Gac pathways [21]. However, the microarray analysis showed that mutation of the spermidine transporter had no effect on transcriptional expression of these global regulatory genes (data not shown). The possibility of posttranscriptional or posttranslational regulation of these global regulatory system by polyamine signals is also not very likely, given that these global systems commonly regulate a wide range of genes encoding various functions [21], [26], whereas the spermidine transporter-mediated signaling mechanism only influences a small set of genes. In this context, it is worthy to note null mutation of the transporter, in addition to down-regulating about 40 T3SS genes, also increased the expression of six genes encoding putative enzymes or proteins. The significance of upregulation and the potential involvement of their gene products in spermidine modulation of T3SS are currently under investigation.

This study has used a combination of genetic, genomic and biochemical approaches to elucidate a novel spermidine transporter-dependent signaling pathway that controls the expression of the T3SS genes of *P. aeruginosa*. Discovery of this signaling pathway has added a new dimension to the known complicated T3SS regulatory networks [21], and may also provide interesting insights into the molecular mechanisms of host-pathogen interactions. Considering that host body fluid contains millimolar ranges of extracellular calcium [1], it becomes highly intriguing how bacterial T3SS system is activated under such a high calcium level during infection. The discovery of this apparent host-pathogen signal communication pathway and the finding that exogenous spermidine alleviates the repression of calcium on T3SS gene expression (Figure 4A, 4B) seem to provide a useful platform to resolve this puzzle. Furthermore, given that many bacterial pathogens appear to have T3SS and the SpuDEFGH-like polyamine transporters [1], [32], [35], [36], this work thus raises an intriguing possibility of potential functional co-evolution of this new signaling pathway with T3SS in other bacterial pathogens.

## Materials and Methods

### Strains and culture conditions

*P. aeruginosa* PAO1 was used as a parental strain for generation of reporter strains and subsequent deletion mutants. The chromosomal integration vector mini-CTX-lacZ [30], and the gene replacement vector pK18mobsacB were used for reporter construction and gene deletion, respectively. The resulted strain- and plasmid-derivatives were described in text and listed with details in Table 2. Unless indicated otherwise, bacteria were routinely grown at 37°C in Luria-Bertani broth (LB) or minimal medium [37]. Following antibiotics were added to medium when necessary: gentamicin (Gm), 30 µg/ml for *P. aeruginosa*, and 5 µg/ml for *E. coli*; tetracycline (Tc), 50 µg/ml for *P. aeruginosa*, and 10 µg/ml for *E. coli*; kanamycin (Km), 800 µg/ml for *P. aeruginosa*, and 100 µg/ml for *E. coli*. Induction of T3SS expression was achieved by supplementing LB medium with the chelating reagent nitrilotiracetic acid (NTA) at a final concentration of 7.5 mM or otherwise indicated.

**Table 2:** Strains and plasmids used in this study*.

Strains or plasmid	Description	Source or reference
*E. coli*		
*DH5α/*λ*pir*	F– f80 d*lacZ*DM15 *endA1hsdR17* (r_k_^−^ m_k_^−^) *supE44 thi-1 gyrA96* D(*lacZYA-argF*), used for plasmid transformation	Gibco
S17-1	res^−^ pro mod^+^ integrated copy of RP4, mob^+^, used for incorporating constructs into *P. aeruginosa*	Laboratory collection
*P. aeruginosa*		
PAO1	Prototrophic laboratory strain	Laboratory collection
ΔspuE	PAO1 with *spuE* being deleted in-frame	This study
ΔspuEH	PAO1 with *spuEFGH* being deleted in-frame	This study
PAO1pClacZ	*lacZ* fused to the *exsCEBA* promoter and integrated at the *attB* site of the PAO1 chromosome	This study
ΔspuEpClacZ	*lacZ* fused to the *exsCEBA* promoter and integrated at the *attB* site of the ΔspuE chromosome	This study
ΔspuEHpClacZ	*lacZ* fused to the *exsCEBA* promoter and integrated at the *attB* site of the ΔspuEH chromosome	This study
*vfr::Tn*	*vfr* disrupted by transposon in strain PAO1pClacZ	This study
*spuE::Tn*	*spuE* disrupted by transposon in strain PAO1pClacZ	This study
*spuF::Tn*	*spuF* disrupted by transposon in strain PAO1pClacZ	This study
*spuH::Tn*	*spuH* disrupted by transposon in strain PAO1pClacZ	This study
*exsA::Tn*	*exsA* disrupted by transposon in strain PAO1pClacZ	This study
PAO1pTlacZ	*lacZ* fused to the *exoT* promoter and integrated at the *attB* site of the PAO1 chromosome	This study
ΔspuEpTlacZ	*lacZ* fused to the *exoT* promoter and integrated at the *attB* site of the ΔspuE chromosome	This study
ΔspuEHpTlacZ	*lacZ* fused to the *exoT* promoter and integrated at the *attB* site of the ΔspuEH chromosome	This study
PAO1lacZ	Promoterless *lacZ* integrated at the *attB* site of the PAO1 chromosome	This study
Plasmids		
pBT20	The mariner transposon mutagenesis vector; Gm^R^ & Ap^R^	Schweizer HP
pDSK519	Broad-host-range cloning vector; IncQ, Km^R^	Laboratory collection
pDSK-spuE	pDSK519 containing *exsA* under the control of *lac* promoter	
pDSK-exsA	pDSK519 containing *exsA* under the control of *lac* promoter	This study
pK18mobsacB	Broad-host-range gene replacement vector; *sacB*, Km^R^	Laboratory collection
pK18GT	pK18mobsacB with Gm^R^ cassette inserted at the NcoI site; Gm^R^	This study
pK18GT-spuEdel	pK18GT containing the *spuE* flanking region with the gene being deleted in frame	This study
pK18GT-spuEHdel	pK18GT containing the *spuEFGH* flanking region with the genes being deleted in frame	This study
mini-CTX-lacZ	Chromosomal integration vector containing a promoterless *lacZ* for construction of transcriptional fusion; Tc^R^	Schweizer HP
mini-CTX-pC-lacZ	mini-CTX-lacZ with the *exsCEBA* promoter fused to *lacZ*; Tc^R^	This study
mini-CTX-pT-lacZ	mini-CTX-lacZ with the *exoT* promoter fused to *lacZ*; Tc^R^	This study

* Symbol: Gm, gentamicin; Ap, ampicillin; Km, kanamycin; Tc, tetracycline.

### DNA manipulation

Plasmid DNA was prepared by QIAprep® Miniprep column (Qiagen). Chromosomal DNA was prepared by MasterPure^TM^ DNA Purification Kit (Epicentre). Restriction enzyme digestion, ligation, and electrophoresis were performed routinely with standard procedures. The nucleotide sequences of PCR-derived constructs were determined with a dye terminator kit and an ABI Prism 3770 sequencer.

### Construction of reporter strains

All the PCR primers used in this study, which were designed based on the genome sequence of *P. aeruginosa* strain PAO1 (http://www.pseudomonas.com) [38]. PCR amplification was carried out with Pfu Turbo polymerase (Promega) using the PAO1 genomic DNA as the template. The promoters of *exsCEBA* and *exoT* were amplified by PCR using the primer pairs pC-F/pC-R (5′-gctctagacggtgatccagtccttc/5′-ggggcgcctcctaaagctc), and pT-F/pT-R (5′-ctcggccgtcgtgttcaagc/5′-aggctgaaggtgcggattcc), and cloned into the integration vector mini-CTX-lacZ [30], [39], respectively. These constructs and the vector control were introduced into *E. coli* S17-1(λpir) and then integrated into the chromosome of *P. aeruginosa* as described previously [30]. The resultant reporter strains were designated as PAO1pClacZ and PAO1pTlacZ, respectively.

Deletion of the internal sequence of *spuE* encoding the peptide from 32-aa to 362-aa and that of *spuEFGH* encoding the amino acid 32 of SpuE till the last amino acid of SpuH were carried out by allelic exchange following the methods described previously [40], except that a new allelic exchange vector pK18mobsacB-Gm was constructed by inserting a gentamicin resistance gene at the NcoI site of the original pK18mobsacB vector for facilitating selection of *P. aeruginosa* transformants. The resulted mutants ΔspuE containing the *spuE* in-frame deletion and ΔspuEH containing the *spuEFGH* in-frame deletion were confirmed by PCR analysis and DNA sequencing.

### Transposon mutagenesis

The marina transposon carried by plasmid pBT20 [41] was used for mutagenesis to screen for the genes involved in the regulation of *exsCEBA* expression. The plasmid was transferred from *E. coli* S17-1(λpir) into the recipient reporter strain PAO1pClacZ by biparental mating at 37°C for 4 h. The mutants were screened on LB agar plates containing Tc and Gm supplemented with 7.5 mM NTA and 50 µg/ml of 5-bromo-4-chloro-3-indolyl-b-D-galactopyranoside (X-gal). The mutants which showed lighter blue than the parental strain PAO1pClacZ were selected for further confirmation. Arbitrary PCR was then used to identify the genes disrupted by transposon insertion as described previously [42]. Briefly, the arbitrary primer AD2 (5′-cangctwsgtntscaa) and the specific primer Gm447 (5′-gtgcaagcagattacggtgacgat) matching the right end of transposon were used for the first round PCR. The primer pair AD2 and the nested specific primer Gm464 (5′-tgacgatcccgcagtggctctc) was then used for the second round PCR. The PCR products were purified using the QIAquick Spin PCR purification kit (Qiagen) and sequenced using the primer Gm487 (5′-atacaaagttgggcatacg). The DNA sequences flanking the transposon insertion were analyzed with the NCBI BLAST server (http://www.ncbi.nlm.nih.gov/blast/) and the Pseudomonas database (http://www.pseudomonas.com/).

### RNA Extraction and microarray analysis

Total RNA samples were isolated from fresh bacterial cultures using the RNeasy mini kit (Qiagen) according to the manufacturer's instructions, and digested with DNase I (Promega) to remove contaminating genomic DNA. The enzyme was then removed by RNeasy column purification. The quantity and purity of RNA were determined by agarose gel electrophoresis and spectrometry. cDNA was synthesized from total RNA samples by using random primers (Invitrogen). SuperScript II (Invitrogen) and biotin-ddUTP was used to label the product according to the protocol from Affymetrix (Affymetrix). Target hybridization, washing and staining were performed following the manufacturer's instructions. GeneChip arrays were scanned with an Affymetrix probe array scanner. The microarray analysis for each bacterial strain was repeated for three times and the data were analyzed using a statistics software MAS-5.0 from Affymetrix.

### Protein isolation and western blotting analysis

Overnight bacterial cultures were inoculated at a 1∶2000 ratio to fresh LB medium supplemented with NTA or spermidine. After incubation at 37°C overnight, the bacterial cultures were chilled on ice for 10 minutes. For each bacterial culture, 10 ml were collected and centrifuged. The supernatants and the bacterial pellets were used for preparation of extracellular proteins and total cellular proteins, respectively. The supernatants were filtered with 0.2 µm syringe filter and precipitated with trichloroacetic acid (TCA) at a final concentration of 10%. The precipitates were pelleted by centrifugation, washed twice with acetone, dried, and re-suspended in SDS sampling buffer. For isolation of total cellular proteins, the bacterial pellets were resuspended in PBS buffer and the cells were broken by sonification. After centrifugation, the supernatants which contain total cellular proteins were kept for further analysis. The protein samples were denatured by boiling for 5 minutes and separated by 10% SDS-PAGE. Western blot analysis was performed following the standard protocols [43].

### Quantitative β-galactosidase assay

Overnight bacterial cultures were diluted 1∶200 to fresh LB medium supplemented with NTA or spermidine as indicated. The growth was continued with shaking at 37°C for 4 h to allow OD_600_ reaching 1.2. β-Galactosidase activity was measured as described [43]. Results were the averages from at least three independent experiments and given as Miller units (MU).

### Culture of *P. areuginosa* with mouse liver extract

Animals were used following the guidelines of the National Advisory Committee for Laboratory Animal Research (NACLAR), with protocols approved by the Institutional Animal Care and Use Committee (IACUC), Singapore. Liver supernatants were prepared using fresh liver from the wild type mouse FVB/N. About 4 g of liver was placed in 10 ml of the calcium-free minimal medium (37) and homogenized by Ultra-Turrax (T25). Liver homogenates were centrifuged at 10,000 rpm for 10 min and supernatants were collected. Bacterial cells were prepared by inoculating overnight bacterial cultures at 1∶200 ratio to 20 ml fresh LB medium and incubated with shaking at 37°C for 4 h to allow OD_600_ reaching about 1.2. The bacterial cells were collected by centrifugation and washed twice with minimal medium before resuspending in 10 ml of minimal medium. The liver supernatants were diluted with minimal medium to desired concentrations and then mixed with the bacterial suspension at the ratio of 1∶1. After incubation at 37°C for 4 h, bacterial cells were harvested by centrifugation. β-Galactosidase activity was measured as described above. Results were the averages from three independent experiments.

### HeLa cell culture and cytotoxicity assay

The cytotoxicity of different bacterial strains was assayed by using HeLa cells. HeLa cells were seeded in 24-well tissue culture plates containing Dulbecco's Modified Eagle Medium (DMEM) and allowed to grow at 37°C for 16 to 18 h to obtain 80 to 90% monolayer confluency (5.0×10^5^ cells/well). Culture supernatants were removed, the monolayer was washed once with PBS buffer. For inoculation, the fresh bacterial cells were resuspended and diluted in DMEM to a concentration about 6×10^7^ CFU per ml. Thereafter, 0.4 ml of the bacterial dilution was applied to the HeLa cell monolayers at a multiplicity of infection (MOI) of 40–50. Cytotoxicity was determined by measuring the release of the cytosolic enzyme lactate dehydrogenase (LDH) into supernatants by using the cytotoxicity detection kit (Roche) at indicated time points post infection.

## Supporting Information

Figure S1 Genetic organization of the spuABCDEFGH locus in wild type strain PAO1 (top), and the corresponding region in the deletion mutants ΔspuE (middle) and ΔspuEH (bottom). The arrow above and the triangle below indicated the direction of transcription and the location of transposon insertion, respectively.
(1.01 MB TIF)

Figure S2 The growth patterns of PAO1 (square), ΔspuE (triangle) and exsA::Tn (circle) in LB medium (open symbol) or in LB medium supplemented with 7.5 mM NTA (solid symbol). Bacteria were grown at 37°C with agitation. Growth was monitored by measuring the optical density of the culture at 600 nm (OD600). Addition of 1 mM spermidine to LB medium with or without 7.5 mM NTA generated almost identical growth curves of PAO1 and its mutants (data not shown).
(0.96 MB TIF)

Figure S3 Deletion of the spermidine synthetic genes speD and speE did not alter the expression of exsCEBA and the secretion of ExoS. (A) Bacteria were grown in LB medium (open bar) or in LB medium supplemented with 7.5 mM NTA (grey bar) and the β-galactosidase activity directed by the promoter of exsCEBA was determined at 4 h after inoculation. The data were the means of at least three replicates±SE. (B) The extra-cellular proteins from P. aeruginosa grown in LB medium supplemented with 7.5 mM NTA were separated by 10% SDS-PAGE. The proteins were transferred onto nitrocellulose membrane and blotted with anti-ExoS antibody.
(1.10 MB TIF)
