# Supplementary figures and images for: Modulation of Bacterial Type III Secretion System by a Spermidine Transporter Dependent Signaling Pathway
Source: PLoS One. 2007 Dec 12;2(12):e1291. doi: 10.1371/journal.pone.0001291 (PMC2110884; doi:10.1371/journal.pone.0001291)

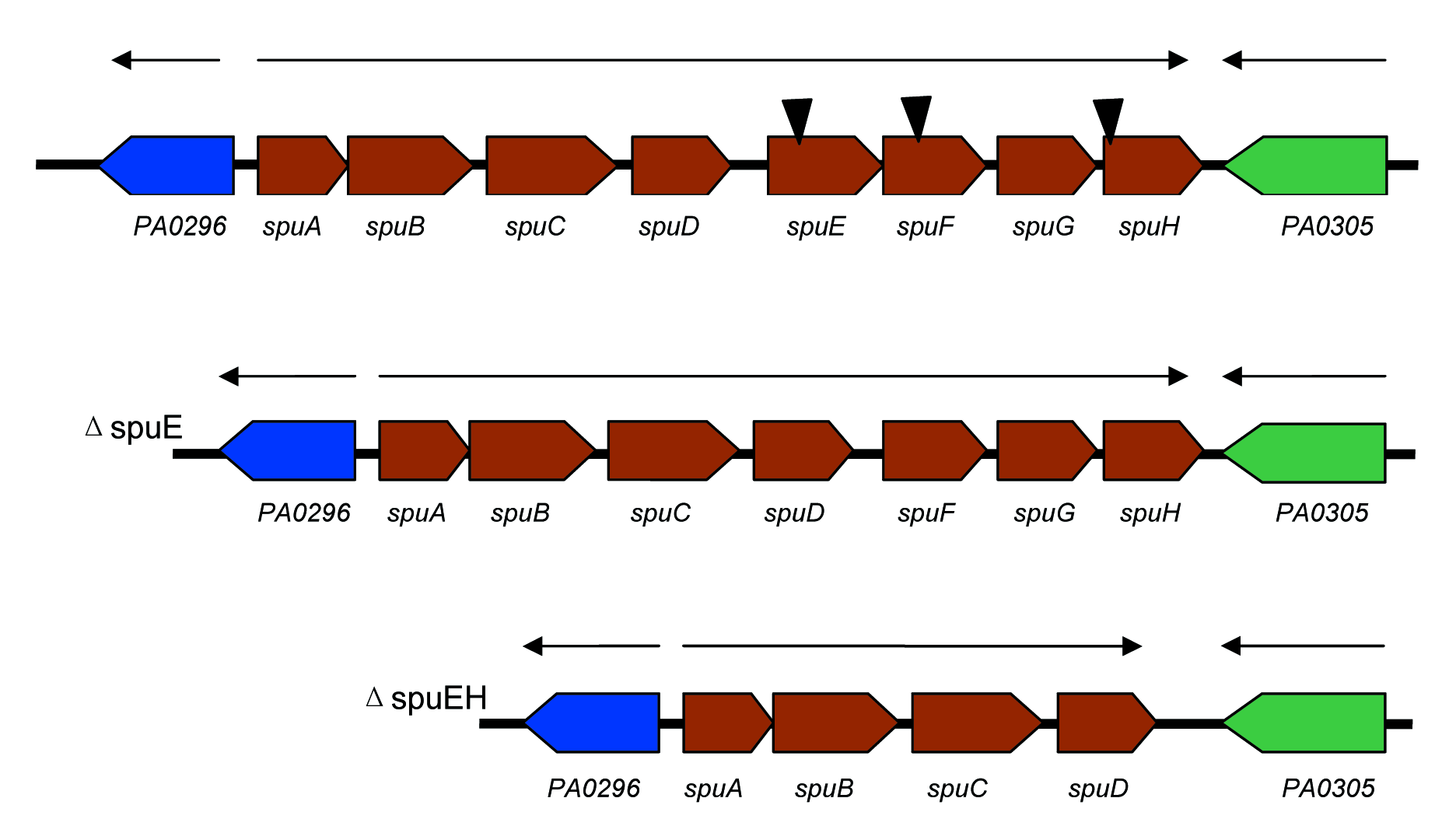

Supplement: Figure S1 — Genetic organization of the spuABCDEFGH locus in wild type strain PAO1 (top), and the corresponding region in the deletion mutants ΔspuE (middle) and ΔspuEH (bottom). The arrow above and the triangle below indicated the direction of transcription and the location of transposon insertion, respectively. (1.01 MB TIF) [file pone.0001291.s001.tif]

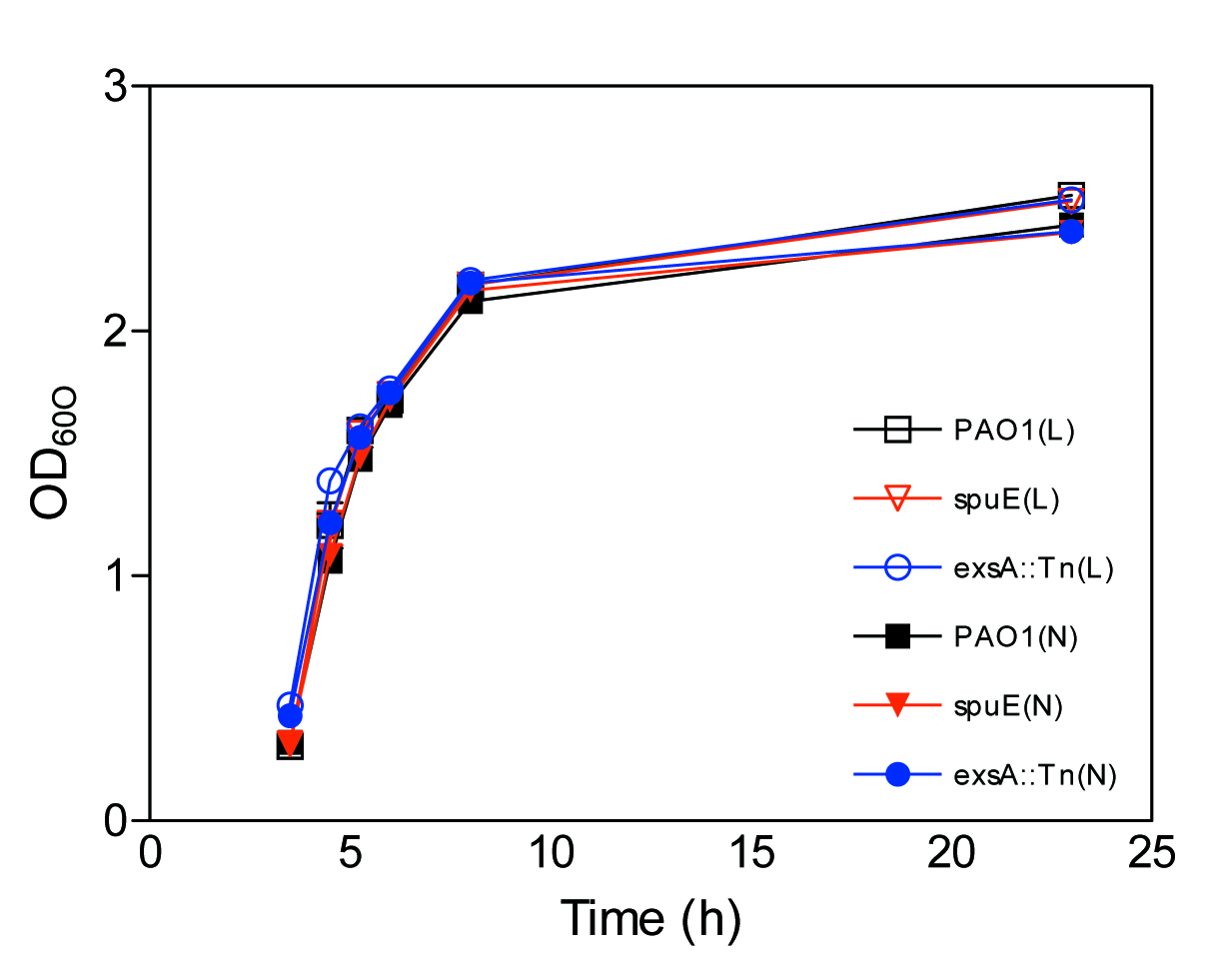

Supplement: Figure S2 — The growth patterns of PAO1 (square), ΔspuE (triangle) and exsA::Tn (circle) in LB medium (open symbol) or in LB medium supplemented with 7.5 mM NTA (solid symbol). Bacteria were grown at 37°C with agitation. Growth was monitored by measuring the optical density of the culture at 600 nm (OD600). Addition of 1 mM spermidine to LB medium with or without 7.5 mM NTA generated almost identical growth curves of PAO1 and its mutants (data not shown). (0.96 MB TIF) [file pone.0001291.s002.tif]

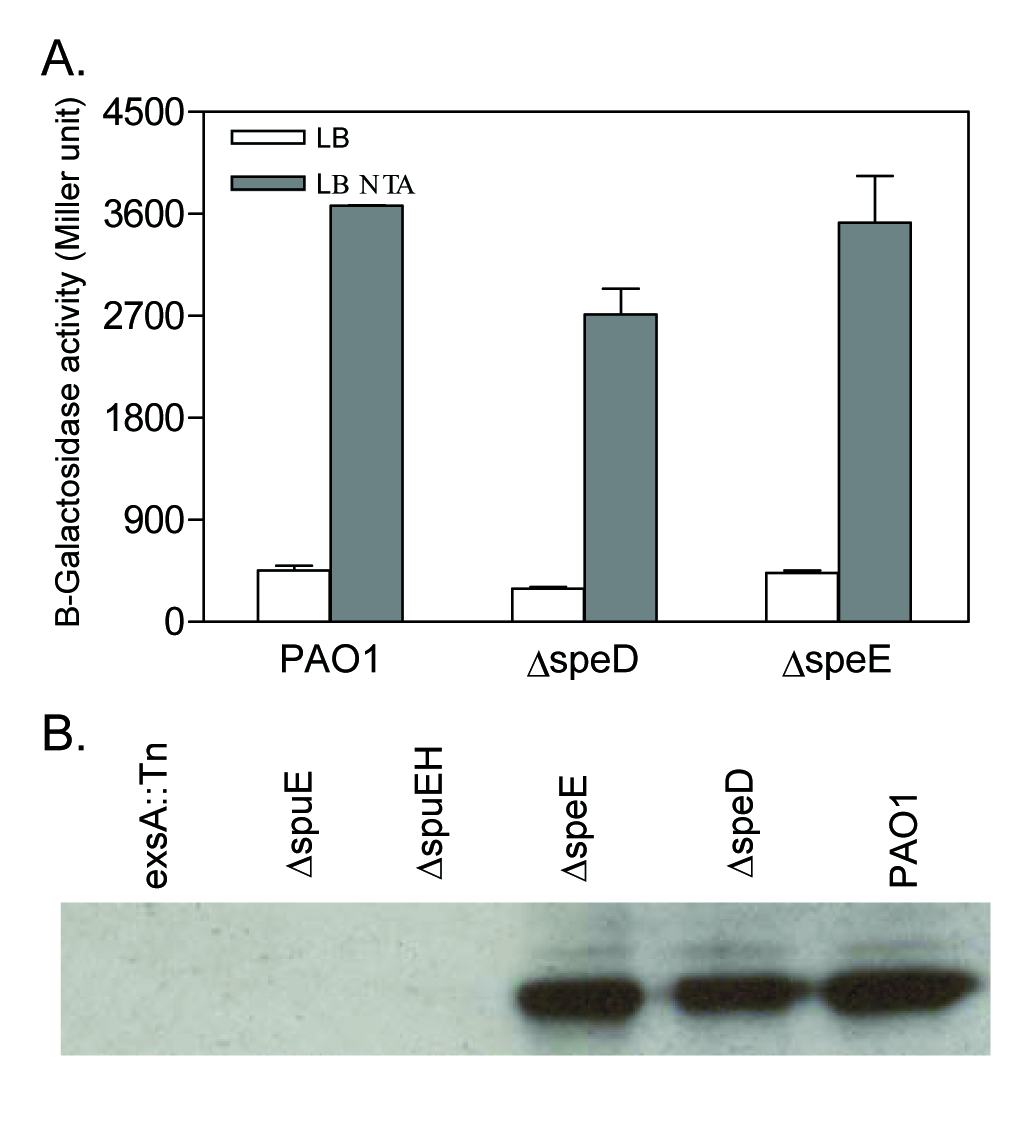

Supplement: Figure S3 — Deletion of the spermidine synthetic genes speD and speE did not alter the expression of exsCEBA and the secretion of ExoS. (A) Bacteria were grown in LB medium (open bar) or in LB medium supplemented with 7.5 mM NTA (grey bar) and the β-galactosidase activity directed by the promoter of exsCEBA was determined at 4 h after inoculation. The data were the means of at least three replicates±SE. (B) The extra-cellular proteins from P. aeruginosa grown in LB medium supplemented with 7.5 mM NTA were separated by 10% SDS-PAGE. The proteins were transferred onto nitrocellulose membrane and blotted with anti-ExoS antibody. (1.10 MB TIF) [file pone.0001291.s003.tif]
